# Hunting, Sale, and Consumption of Bushmeat Killed by Lead-Based Ammunition in Benin

**DOI:** 10.3390/ijerph15061140

**Published:** 2018-06-01

**Authors:** Shukrullah Ahmadi, Suzanne Maman, Roméo Zoumenou, Achille Massougbodji, Michel Cot, Philippe Glorennec, Florence Bodeau-Livinec

**Affiliations:** 1 EHESP, F-35000 Rennes, France; philippe.glorennec@ehesp.fr (P.G.); florence.bodeau-livinec@ehesp.fr (F.B.-L.); 2 Obstetrical, Perinatal, and Pediatric Epidemiology Team, Centre of Research in Epidemiology and Statistics Sorbonne Paris Cité (U1153), INSERM, Paris Descartes University, 75020 Paris, France; 3 Department of Health Behavior, Gillings School of Global Public Health, University of North Carolina at Chapel Hill, Chapel Hill, NC 27599, USA; maman@email.unc.edu; 4 Faculté des Sciences de la Santé, Université d’Abomey-Calavi, Cotonou, Benin; zoumenour@yahoo.fr (R.Z.); massougbodjiachille@yahoo.fr (A.M.); 5 MERIT (Mère et Enfant Face aux Infections Tropicales)—UMR 216, Institut de Recherche pour le Développement (IRD), Université Paris Descartes, 75006 Paris, France; michel.cot@ird.fr; 6 Centre de Recherches des Cordeliers, Université Pierre et Marie Curie, 75006 Paris, France; 7 Faculté de Pharmacie, Université Paris Descartes, 75006 Paris, France; 8 Inserm, Irset (Institut de Recherche en Santé, Environnement et Travail)—UMR_S 1085, F-35000 Rennes, France

**Keywords:** Africa, lead exposure, sources, dietary metal exposure, game meat, health

## Abstract

Human consumption of animal meat killed by lead ammunition has been reported as a risk factor for elevated blood lead levels. However, little is known about how meat killed by lead ammunition is hunted, prepared, sold, and consumed. We explored the process from hunting to consumption within communities in Benin from the perspective of preventive measures. We conducted 38 semi-structured interviews with hunters (*n* = 9) and sellers (*n* = 8) of bushmeat and families (*n* = 21) as consumers of bushmeat killed by lead ammunition. Data were transcribed, translated, and coded for analysis. We conducted content analysis to identify and describe key themes and processes from hunting to consumption. Many hunters (*n* = 7/9) used lead-based ammunition. After the meat is hunted, market sellers often buy it directly from the hunters. Amongst the hunters and sellers, few (*n* = 4/17) acknowledged removing the meat impacted by lead shot prior to sale. Many families (*n* = 15/21) mentioned consumption of the hunted bushmeat. The meat is cooked before sharing with children. Many families (*n* = 19/21) mentioned they look for the remains of the lead shot or remove the meat impacted by the shot. The finding suggests that hunting, sale, and consumption of bushmeat killed by lead ammunition are well-known practices in Allada, Benin. The bushmeat often hunted illegally with lead shot is sold in the markets and eventually consumed by families who attempt to clean the meat impacted by the lead shot before cooking it.

## 1. Introduction

The term bushmeat applies to meat derived from wild or forest animals including mammals, reptiles, amphibians, and birds hunted for food in tropical forests [1]. Bushmeat represents an important source of animal protein for humans in tropical Africa [2] and is increasingly becoming a preferred meat over domestic meat for local populations in many African countries [1,3]. It is estimated that 30–85% of daily protein intake of Africans comes from this meat [1], consequently contributing significantly to food security in many African regions [4]. In addition, unemployment and poverty in African rural communities are common, and the possibility of quick income generation from selling bushmeat is a common incentive for bushmeat hunting [4]. Hunters with part-time or seasonal employment devote more time to hunting than those with full-time jobs [5]. In most African countries, including Benin, hunting of bushmeat is regulated by relevant laws and licensing systems that generally aim to restrict hunting (for example, by prohibiting hunting in certain protected areas and restricting hunting to certain species and at certain times of year) [4]. However, illegal hunting of bushmeat is often committed due to inadequate law enforcement and weak penal systems [4]. Bushmeat hunters in Africa may also employ a variety of non-ammunition hunting methods including, but not confined to, snares, traps, fire, nets, and dogs. Most of these methods are illegal in many African countries, including Benin. In many regions, firearms, such as shotguns, are used, while automatic firearms are rarely used [4].

The consumption of bushmeat received growing attention during the 2013–2016 Ebola virus disease (EVD) epidemic in West Africa [6]. Consumption or handling of bushmeat infected with Ebola virus, is a known risk factor for infection transmission to humans [7,8,9]. This led to a ban and stigmatization of bushmeat in many West African countries [6,10]. Briefly, one of the initial public health measures implemented to control the West African EVD outbreak was to ban hunting, sale, and consumption of bushmeat [10]. As a result, a reduction of bushmeat consumption during the outbreak has been reported in a few West African countries [2,6].

Public health concerns have also emerged as several studies [11,12,13,14,15,16] reported an association between consumption of meat hunted with lead (Pb) ammunition and Pb exposure in humans. Many studies on the consumption of wild meat hunted with Pb ammunition have been undertaken outside Africa [14,15,17,18,19,20,21,22,23,24,25]. Pb exposure occurs from the consumption of hunted meat contaminated with the residues of Pb fragments from hunting ammunition [13,14,15,17,18,26,27]. Hunted meat is contaminated with small Pb fragments as Pb shot travels through the flesh of hunted animal [19]. Pb in the meat is bioavailable [27] and becomes more bioaccessible after cooking it [19]. These deeply-rooted fragments are considered one of the most important sources of dietary Pb exposure in communities that substantially rely on the consumption of this type of meat [19].

Pb exerts its harmful effects on multiple body systems, including the neurologic, hematologic, gastrointestinal, cardiovascular, and renal systems [28]. Children are the most vulnerable group to the neurotoxic effects of Pb due to their developing brain [11,29] even at low levels of exposure [20,28,30]. Hunters may also be exposed to Pb [26] through certain behaviors related to hunting: consumption of Pb shot contaminated meat [21,31], inhalation of lead dust during the recharge of the firearm, homemade fabrication of lead ammunition, and preparation of carcasses hunted by Pb shot [21]. The WHO estimates that overall Pb exposure was responsible for 143,000 deaths in 2004 and 0.6% of the global burden of disease expressed in disability-adjusted life years, or DALYs, mainly considering cardiovascular outcomes and mild mental retardation attributable to lead exposure [32,33]. A recent study has estimated that about 400,000 deaths in the adult population in the USA are attributable to low-level Pb exposure (5 μg/dL) every year, making Pb exposure an important risk factor of mortality [34].

Practices of the hunting, sale, and consumption of bushmeat killed by Pb ammunition in Africa have not been well described in the literature. The few published papers that have either studied or described bushmeat consumption in an African context [3,35,36,37,38], and particularly in Benin [3], have studied the issue in relation to the wildlife conservation and food security [3,38] but not in relation to potential Pb exposure in humans.

As part of a larger study on Pb exposure in children [11], we aimed to explore the selective hunting, sale, and consumption practices of bushmeat killed by Pb ammunition. The purpose was to generate meaningful information that could be helpful in developing future preventive programs to reduce the risk of Pb exposure in Benin and more broadly in Sub-Saharan Africa (SSA).

## 2. Materials and Methods

### 2.1. Background on Parent Study

The parent study, the TOVI study (in Fon language *Tovi* means child from the country), investigated Pb exposure and potential sources of Pb for 685 one-year-old children in Benin between May 2011 and May 2013 [11]. This study found that 38.8% of 1–2-year-old children consumed this type of meat in their families. Furthermore, 58% of infants and 44% of mothers presented elevated blood lead levels (BLL) i.e., higher than 50 µg/L (5 μg/dL). The study also identified consumption of animal meat killed by ammunition associated with elevated BLL in one-year-old children. Children from the TOVI cohort are followed at six years of age in the ongoing EXPLORE study (2016–2018) to reassess BLL, potential sources, and neurocognitive development.

### 2.2. Study Design

A qualitative design, using semi-structured interviews, was developed to explore the hunting, sale, and consumption of bushmeat. A flexible qualitative design was anticipated to be more practical to help explore the practices and processes involved in the acquisition, preparation and consumption of bushmeat. A qualitative approach was critical given the potential sensitivity attached to the hunting, sale and consumption of bushmeat [10].

### 2.3. Study Subjects

The study took place in Allada, a district in the south of Benin. We sampled 38 individuals including 21 families (bushmeat consumers), nine bushmeat hunters, and eight bushmeat sellers. Families, usually a relative accompanying the child during the child’s scheduled medical visit, were selected from the ongoing EXPLORE study. The children did not have any input in the interviews. Seventeen family respondents were the mother of the index child, and two were grandmothers of the child. These families were scheduled within the EXPLORE project at the health center between March and April 2017. They were interviewed one day before their visit. On the other hand, bushmeat hunters and sellers were recruited through snowball sampling.

### 2.4. Interview Procedures

Semi-structured interviews were conducted. The interview guide (see Supplementary Material) outlined the topics to be explored during the interview. The guide contained open-ended questions related to hunting (for hunters), sale (for sellers), meat preparation (for all the participants), cooking (for sellers and families), and meat consumption practices (for families). Fieldwork with hunters and sellers was carried out in the center of Sekou (a town under the administrative jurisdiction of Allada) and four villages of Sekou. Interviews with hunters and sellers were carried out primarily at their personal residences in Sekou, and one market seller was interviewed in his restaurant.

None of the participants refused to be interviewed. A detailed description of the study was given before participants provided their consent and were interviewed. No personally identifying information was collected. Interviews were conducted either in French (the official language in Benin) or in Fon (one of the most important local languages spoken in the South of the country). Interviews were conducted by the field researchers (SA, RZ, and FBL) with the assistance of a local field assistant who was trained in qualitative data collection and was briefed about the study purpose. It was emphasized that participants did not have to respond to questions, could end the interview at any time without consequences, and their responses would be recorded anonymously. The interviews were recorded with an audio recorder device and were later translated and transcribed into English by one of the field researchers (SA). Field notes were also taken during the interviews which were incorporated into the data for analysis. The semi-structured interviews took place between 11 March 2017 and 15 April 2017. The study obtained authorization from the ethical committee from the Institut des Sciences Biomédicales Appliquées (ISBA) in Benin (N° 87).

### 2.5. Data Analysis

Content analysis was undertaken to find meanings from the content of the text data [39]. The interview data were coded using a combination of inductive and deductive codes. The codes, usually a word or phrase, were used to summarize or condense the interview data. Coded data were reviewed to identify emerging patterns within and across topics. Similar codes were clustered together to develop categories e.g., bushmeat consumption and bushmeat preparation. Quotes were selected that represented patterns in the data. The frequency of responses within specific categories was calculated. When describing reports, the qualifier “few” is used when less than one quarter of participants mentioned a specific practice and “many” when more than three-quarters mentioned it. Transcription, coding, and analyses of text data were conducted in a qualitative data analysis Software: NVivo Pro version 11 (QSR International, Melbourne, Australia).

## 3. Results

Thirty-eight semi-structured interviews were undertaken, including 21 family members, nine bushmeat hunters, and eight bushmeat sellers. The average ages of respondents were 38.6 years (families), 48.4 years (hunters), and 44.5 years (sellers). All hunters were male, and some sellers were female (*n* = 5/8). More than half of the families interviewed were agriculturalists, while others were involved in small-scale village business such as selling of goods (cloth, food, and gasoline). All hunters held a second job in addition to hunting. On average, hunters and sellers had 26.6 years (range = 15–40 years) and 16.4 years (range = 7–30 years) of experience in hunting and sale, respectively.

Based on the objectives of this paper, we have organized the presentation of results to reflect the bushmeat hunting-to-consumption process.

### 3.1. Hunting of Bushmeat

Hunters are the first people who make bushmeat available by hunting wild animals in nearby bushes or distant jungles. Animals often hunted include, but are not confined to, small birds (e.g., partridge), rabbits, rodents (e.g., agouti, squirrel, and rats), and other big animals (e.g., antelope, deer species, and elephants). Rats are less often hunted due to fear of contracting EVD and Lassa fever. Hunters mentioned that it is relatively easier to hunt small game animals such as small birds and rabbits in the nearby bush close to neighborhoods than hunting big animals such as antelope, deer, and elephant in distant jungles. Many interviewed hunters started hunting from a young age, and many had a second occupation besides hunting. In addition, none of the hunters cited possessing a hunting permit. Some hunters described hunting at night time to increase their chances of hunting more animals. A hunter described his past and current hunting experience and the types of animals hunted.

I have been doing this since I was very young. My father used to do it. I saw my father with his gun. I grew up hunting in my family. […]. We did hunting together [with my father]. We hunted rabbits, agouti, small rodents, squirrels, [and] rats. Based on this [past experience of growing up in a family who hunted small animals] we started hunting. Today there are big wild animals. We hunt the big animals, for example, deer, antelopes. They are not a lot here, therefore, we go far from here [into the forests]. [...]. We find big animals there. Here [in the neighborhood] we hunt agouti and other small animals. […]. During the day we do hunting of the day and at night we do the night hunting [...]. Because there are nocturnal animals that come out uniquely at night (ID-01H).

The frequency of hunting varied among hunters and depended on several factors including climatic conditions (e.g., dry, rainy), availability of animals, and their personal availability. The role of season and availability of hunters is also important. In most cases, hunters may not prefer hunting if busy in another occupational activity e.g., agriculture. Therefore, depending on these factors, many hunters reported going hunting at least once a week. One hunter elaborated that he accommodated hunting activity in his schedule depending on the availability of his main occupation:

I hunt any time, any moment, but when I find the construction work [construction of buildings in the village as a primary paid job] then I don’t hunt otherwise I am always in the jungle to hunt [wild] animals (ID-04H).

In terms of ammunition use, many hunters (*n* = 7/9) described that they use shotguns with shotgun shells containing multiple Pb shots for hunting bushmeat. Every hunter owned their own shotguns that they had usually owned for a long time, often inherited from their forefathers. Due to the lack of hunting permits, hunters mainly acquired their shotgun shells by illegally purchasing them. According to hunters, it is difficult to purchase ammunition without a valid hunting license. One hunter elaborated his experience of purchasing shotgun shells through informal networks:

I do not have a hunting permit but I hunt under the [informal] collaboration of someone who has a [valid] permit from whom I can buy [hunting] ammunition. I tried many times to get a hunting license but I could not get one. The authorities do not give it easily. So I buy the cartridges [shotgun shells] from the person who has a license to sale [shotgun shells] and hunts [wild animals]. They will sell it to you if you don’t misuse it. You do not use it for wrong purposes like theft or killing someone. It is much regulated. Once I buy the cartridge [shotgun shells] I am not allowed to sell them to someone else. Everybody [without a valid permit] cannot buy it, it’s not like you go there and they will sell it to you. You need to know someone who could help you (ID-O2H).

In terms of removal of Pb shots from the animal meat, hunters mentioned that they generally do not look for or remove the Pb shot from the meat. However, a few (*n* = 2/9) mentioned they had searched for the Pb shots but they had difficulty detecting them. They felt that the Pb shots would be more likely to be detected during the consumption of bushmeat. One hunter described his preparation practice as follows:

When we prepare the meat we take out the skin and cut the meat into pieces, we do not remove the part where the bullet [shot] has entered. Sometimes when we eat the meat [after cooking] we find it [lead fragment] during consumption. But if the animal is wounded extremely, we remove the wound where the bullet [shot] has entered otherwise we keep it [wound] for consumption (ID-04H).

### 3.2. Sale of Bushmeat

In the hunting-to-consumption process, the next level of individuals who have contact with the bushmeat is the market sellers. Most of the interviewed sellers mentioned selling bushmeat killed by Pb-based ammunition. The market sellers, most often women in this study, typically buy the meat directly from hunters and take it to restaurants or to markets to sell it to bushmeat consumers. In a few cases, the seller is a family member, e.g., wife of the hunter, who sells the hunted meat in markets. A market seller, with 20 years of work in selling bushmeat, described his work:

I have friends who do hunting, I go to their place to buy their hunted meat and resell it to the consumers in restaurants and hotels. I do not prepare it myself, I just buy it from the hunters and sell it like that [raw and unprepared] to the clients who will prepare it [themselves] and sell it [to the bushmeat consumers] (ID-05S).

The sale of bushmeat was described as an activity that is well known in the community and had existed for a long period of time in the region. Sellers mentioned that the demand for this meat is high and people want to buy it despite several factors, such as bad season. A market seller who owned a restaurant described his work:

I was born into this business, my mother also sold bushmeat. I learned it from her and started selling bushmeat at a very early age. […]. There are many people that are looking for this type of meat. [...]. I extend my work also in Cotonou and Allada. […]. I have other friends as well who prepare it for sale. The business is quite good. I have clients in Cotonou, Allada and even in Sekou who come here and pay me for buying it (ID-03S).

Field observation also demonstrated that there were small restaurants where the meat was cooked and sold to consumers. During the field observation, we observed that bushmeat was sold in ordinary food markets as street food. Many sellers mentioned that they sell bushmeat several times per week and some sell it daily, depending on the availability of bushmeat by the hunters from whom they buy it. However, few (*n* = 2/8) sellers mentioned discontinuing the sale of bushmeat due to the outbreak of EVD and Lassa as narrated by one market seller who used to sell bushmeat for 12 years:

I used to sell it every day but due to the epidemic of Ebola and Lassa we left it, now I sell nothing when they talked about rats and these diseases we had to stop everything. […]. It has been at least nine months. When there was one case of Lassa in the North people stopped it. Now hunters hardly find it. Now we consume pork and mutton (ID-07S).

Preparation of the meat is an important step before it is sold to consumers. However, many (*n* = 6/8) market sellers mentioned that they do not remove the part affected by the Pb shot and do not look for the Pb shot before selling it. However, a few market sellers, especially those who sell it in the restaurants, mentioned that cleaning the part affected by the Pb shot is an important part of preparation. This is stated by one market seller with 20 years of working experience who also owned a small restaurant:

When we buy it from the hunters I prepare it here; my wife also prepares it. It should be cooked well with spices [...] it is that [spices] which attract the clients, they will eat it well [with satisfaction]. If it’s not prepared well they will not eat it […] when they [women who prepare the meat] take out the skin, the place where the meat is hit by the bullet [shot], we take that part out because if it is not prepared well and if the consumers find the shot [lead] there (in the meat), they will not come to eat at your place anymore. We prepare that part very well otherwise the consumers will not buy it (ID-03S).

### 3.3. Bushmeat Consumption

Many of the families (*n* = 15/21) mentioned that they currently consume bushmeat killed by ammunition along with other members of their family. Among these families, 10 families reported a consumption frequency of at least once per month. All families mentioned that the consumption was shared with children in the family. The types of meat most commonly consumed included rabbit, agouti, and small birds as described by one mother:

Yes, we sometimes eat agoutis, partridge, [and] rabbit. All the family members [including children] eat it [bushmeat] […] now we do not eat [bushmeat] a lot because I don’t find time to go and kill [wild] animals so when I get it [bushmeat] from the people who hunt them [wild animals], I buy it and it’s [bushmeat consumption] not very frequent […] We eat it less than 2 times per month (ID-04F).

However, several families (*n* = 10/15) mentioned that their consumption frequency has reduced over the last few years for different reasons, most commonly due to the unavailability of bushmeat and EVD outbreak, although this reduction could not be quantified. All families (*n* = 6/21) who did not consume bushmeat currently mentioned bushmeat consumption in the past years.

Families may either purchase bushmeat directly from a hunter or a bushmeat seller or they may acquire it from a family member who hunts wild animals, as described by one mother:

Sometimes we eat the meat of partridge and agouti [rat] killed through hunting by my husband. Sometimes when my husband went out hunting he hunted birds, my husband brought them home, [and] I prepared and ate them. The children also eat it […]. When my husband doesn’t find time to go hunting we find it through other people who sell it. When my husband [after purchasing the wild animal] brings it home, I prepare it and we eat it [together]. […]. Sometimes we eat it once a month (ID-02F).

All families interviewed mentioned that they cook the meat before consuming it. Families most often boiled the meat. Other cooking methods cited included frying and grilling the meat. A mother described her cooking method as following:

I put water [in a pot] on fire to boil it well, then add meat in the water. Next is removing the skin, Umm [next] cutting the meat in to small pieces after chopping the whole meat and then I look for any bad elements inside, if I find something bad I remove it, after cutting in to small pieces I mix some spices and then heat it very well so no there is no bad effects on health (ID-10F).

Many of the interviewed family members (*n*= 19/21) mentioned that before cooking the meat they look for the Pb shot or remove the wounded meat area where the Pb shot enters. They also mentioned that sometimes they manually look for the Pb shot in the meat before cooking it. They acknowledged that it is sometimes difficult to detect the shots or Pb fragments when they search for them in the meat. Some commonly cited reasons for removal of the Pb fragments were health reasons, stomach ache, the perceived appearance of the metal (lead) in the blood, and intestinal damage caused by the metal ingestion. One mother described her preparation method as following:

*After removing the skin, I remove the part affected by the bullet [shot], I separate it and put it in the highly heated oil in order to prepare [cook] it well and get rid of any bad effect of the bullet [shot] on the meat (ID-14F*).

## 4. Discussion

Findings suggest that hunters and market sellers are the individuals who make bushmeat available for consumption. Hunting of bushmeat occurs with Pb-based ammunition without hunting permits and thus hunting occurs illegally. The sale of bushmeat occurs through market sellers who make the meat available in markets where families may purchase it. Families may also acquire bushmeat directly from hunters. It can be acquired either by purchasing it or acquiring it freely if the meat is hunted by a family member. Hunters hunt bushmeat at least once per week, while market sellers sell the meat several times per week. Many families mentioned current consumption of bushmeat killed by Pb-based ammunition. Ten of these families described their consumption once per month or more. The findings, therefore, suggest that the hunting, sale, and consumption of bushmeat killed by Pb-based ammunition are common and well-known in this setting.

Considering the published evidence on the risk of lead exposure due to consumption of meat hunted with lead, we put forward several preventive measures. However, the feasibility of these measures, in an African context, should be further investigated.

### 4.1. Controlling Bushmeat Hunting and Sale

In this study, no hunter possessed a hunting permit. The enforcement of laws that forbid the trade and consumption of bushmeat is crucial for reducing the demand for bushmeat [40]. However, illegal hunting and trade could continue due to inadequate law enforcement and weak penal systems [4]. Local control and ban on bushmeat hunting, sale, and consumption commonly intended for other purposes such as conservation of wildlife and prevention of infectious diseases, like EVD [10], may discourage some people from consuming bushmeat, but such legal sanctions should be deployed with caution as it could have unintended social and political repercussions [10]. Additionally, given the difficult economic situation of rural communities that heavily rely on bushmeat as a source of nutrition or income, alternative protein sources have to be facilitated [2,4,41]. In terms of market regulations, changing bushmeat prices could also impact the demand for bushmeat [1,42]. A study undertaken in Tanzania suggested that increasing the price of bushmeat, to reduce the bushmeat supply, could reduce bushmeat consumption [42].

### 4.2. Replacement of Pb Ammunition

The use of non-Pb ammunition can remove the risk of Pb contamination of hunted meat [16]. Therefore, replacing Pb ammunition with non-Pb for hunting could be an effective strategy [21,27,43]. Use of Pb-based ammunition should be controlled, and non-Pb ammunition should be promoted [22]. In general, studies outside Africa have shown that the cost of replacing non-Pb bullets is minimal [27,43,44]. A study that assessed the retail prices of equivalent lead-free and lead-core bullets in the USA and Europe found no disparity in cost for most popular calibers [45]. In addition, evidence suggests that such measures can be effective. In 1999, when it was pointed out that Pb ammunition was a likely source of Pb exposure in Inuit populations of Nunavik, Canada, a local control and ban on the use of Pb shot ammunition in this region showed a drastic decrease in Pb exposure and a decrease in the availability of Pb shot ammunition in stores of this region [21].

### 4.3. Removal of the Meat around the Shot Channel

In terms of bushmeat preparation, many hunters and sellers mentioned that they do not attempt to remove the meat around the shot channel—a precautionary measure to reduce Pb exposure in the meat. In fact, for a few of them, a good presentation of the meat (absence of visible wound around the shot channel) is important in order to get it sold. This suggests the meat around the shot channel may only be removed if largely visible wounds are present, which could have a negative effect on the presentation of the meat to potential customers. On the other hand, many families mentioned that they search for any visible Pb shots and clean or remove the meat impacted by the shot. However, it could not be assessed what portion of the meat is cleaned and removed by the families in this study. Although there is a lack of consensus in the literature regarding the efficacy of this precautionary measure [16], based on a Norwegian study, it is necessary that the meat along the bullet channel of big animals is removed in a radius of 30 cm (60 cm diameter) [16]. In theory, it is possible that discarding the wound affected by Pb shot could reduce exposure to Pb. However, Pb bullets break into hundreds of tiny fragments as they pass through animal meat [18], and therefore the entire removal of all small Pb fragments is almost impossible [19] as long as the use of Pb ammunition is continued [29]. In addition, the removal of meat along the bullet channel in a radius of 30 cm may not be applicable to small animals, e.g., birds.

### 4.4. Reducing Bushmeat Consumption and Promoting Alternative Food Sources

Amongst the families who reported current consumption of bushmeat, many reported a frequency of at least once per month. A local study published in 2004 and undertaken amongst residents (*n* = 126) living near the Lama forest in the South of Benin found that 82% of people preferred bushmeat over fish and meat from domestic animals as a principal source of protein [3]. However, bushmeat was found to be less commonly consumed as compared to fish (34% vs. 60%) and constituted about 1/3 of animal protein consumed [3]. Studies have reported that a consumption frequency of (cervid) game meat once per month or more was associated with approximately 31% increase in blood Pb concentrations [16].

In terms of preventive measures, a Norwegian study recommends that children, pregnant women, and women of reproductive age should not consume meat killed by Pb-based ammunition more than once per month [16]. It is therefore important to spread awareness [27] among families, hunters, and sellers about the risk of Pb exposure from the consumption of bushmeat killed by Pb based-ammunition.

It is also important to promote alternative food sources [2] that can minimize reliance on bushmeat [2,4,46]. For example, promotion of domestic animals, which is already in practice in this region [3], can encourage consumption of meat from domestic animals as a substitute to hunted bushmeat. However, the negative environmental impact from increased livestock production must be properly managed [4,43]. Moreover, many studies have shown that fish represents an important alternative protein source to bushmeat [46,47,48], but promoting fish as a direct replacement to bushmeat requires improved management of domestic fisheries in order to ensure sustainability of fish stocks [42,48].

### 4.5. Strengths and Limitation

To our knowledge, this is one of the first studies to explore bushmeat consumption with special reference to Pb exposure in West Africa. The qualitative design of the study helped us to explore a potentially sensitive topic. This study also discussed several recommendations on the issue that could be explored further as preventative measures. This study was undertaken in the context of the post-EVD outbreak, which could have influenced participants’ responses (on bushmeat hunting, sale, preparation, and consumption practices) reported in this study. Although attempts in the design and data collection phase of the study such as indirect questioning methods [49] and asking sensitive questions toward the end [50] were applied, we cannot completely rule out social desirability bias. In addition, frequencies (e.g., bushmeat consumption) reported here should be interpreted with caution as we did not intend to sample representatively. Further epidemiological and quantitative findings on the topic will be studied in the ongoing EXPLORE study. Furthermore, we did not assess the quantity of meat consumed and the lead content in the meat. 

### 4.6. Research Needs

Further exposure, risk, and perception assessment of the population due to hunting, manipulation, and consumption of bushmeat killed by Pb ammunition would help us to understand the extent of the risk of Pb exposure linked with bushmeat killed by Pb ammunition. There is a lack of information about the scale, distribution, trends, and perception of Pb exposure associated with consumption of bushmeat killed by Pb ammunition in this population. This lack of information undermines efforts to mobilize health authorities to develop coordinated, inter-sectoral responses to address the issue. Additionally, there is a need to assess the efficacy and feasibility of the above-mentioned recommendations in an African context that, to our knowledge, have not been studied before with specific reference to Pb exposure. Finally, Pb exposure has different sources. Studying other local sources of Pb exposure may improve our understanding of different sources in this region.

## 5. Conclusions

This study explored the hunting, sale, and consumption of bushmeat with special reference to the risk of Pb exposure, which is a neglected public health issue in Sub-Saharan Africa. Our findings suggest that bushmeat is often illegally hunted with Pb-based ammunition and sold either in markets or directly to the consumers. Only families who consume bushmeat, clean the part of the meat impacted by the lead shot before cooking. Their consumption frequency of 1–2 times per month may certainly pose a risk for Pb exposure. As there is no safe level of Pb exposure in humans, serious attention of the public health authorities and researchers are required in this regard. Failure to address the problem could have consequences on the health of the population, particularly young children who share consumption of the Pb-contaminated meat in their families. Although this study adds evidence to the limited existing body of research on the hunting, sale, and consumption of bushmeat killed by Pb ammunition in this region, further research may be required to assess and validate Pb exposure from the consumption of bushmeat killed by Pb-based ammunition in this region.

## Supplementary Materials

The following are available online at http://www.mdpi.com/1660-4601/15/6/1140/s1, Interview guide and Qualitative data statement.
