# Supplementary material for: Hunting, Sale, and Consumption of Bushmeat Killed by Lead-Based Ammunition in Benin
Source: Int J Environ Res Public Health. 2018 Jun 1;15(6):1140. doi: 10.3390/ijerph15061140 (PMC6025093; doi:10.3390/ijerph15061140)
Supplement: Supplementary file 1 [file ijerph-15-01140-s001.pdf]

INTERVIEW GUIDE

Hunting, Sale, and Consumption of Bushmeat Killed by Lead Ammunition

Italics typeface: notes for interviewer
Questions in parentheses: additional probing questions

Introduce yourself and explain the purpose of the interview. Remind respondent that the answers are anonymous and that he/she can skip questions and/or stop the interview at any moment.

A. Personnel data

1. Profession
2. Age
3. Sex
4. Education

B. For people who hunt

Try and have interviewee bring up bushmeat without you mentioning it.

1. Could you tell me about your hunting?
2. Could you tell us about your hunting experience? Since when you have been hunting?
    a. Could you describe how you hunt?
    b. What species do you hunt?
2. What do you do with the meat soon after hunting it? *Probe for preparation of the meat.*
    a. Do you sell or eat what you hunt? With whom?
    b. Can you tell me how you prepare the meat after hunting?
    c. What do you do to the meat wounded by the bullet? (Do you clean it or discard it? How? Why?)
3. What kind of weapons do you use? Do you use ammunition?
    a. What kind of hunting gun do you use?
    b. What kind of bullets do you use for hunting? (What are they made
up
    of? Do you use bullets made up of lead?)
    c. How do you acquire ammunition for hunting animals? (Where do
you
    get bullets for hunting? From whom do you get bullets? Do you purchase bullets? Is it easy to purchase them?)
4. How much time do you spend hunting in a month? *Probe for frequency of hunting.*
5. Do you need permission from local authorities to hunt? *Probe for hunting permit.*
    a. (Do you need to have a hunting permit? Do you have one?)

C. For people who sell bushmeat

Try and have interviewee bring up bushmeat without you mentioning it.

1. Could you tell us what species you sell?
    a. Where do you sell it?
    b. From who do you obtain it?
    c. How have these animals been killed? (By trap or by ammunition?)

   d. Can you describe how you sell the meat to consumers? (Do you sell it yourself?)
2. Do people here buy bushmeat?
3. How much time do you spend selling bushmeat in a month? *Probe for frequency of sale.*
4. Can you tell me how you usually prepare this meat before selling?
   a. (Do you cook it? Can you describe how you cook the bushmeat?)
   b. What do you do to the meat wounded by the bullet?
   c. (Do you clean the meat wounded by the bullet? Or discard it? How? Why?)

D. Families/bushmeat consumers

Try and have interviewee bring up bushmeat without you mentioning it.

1. What kinds of animal meat do you eat usually?
   a. What about consumption of wild meat/bushmeat?
   b. How has this meat been killed/hunted? (Is it killed by ammunition?)
   c. Do you consume it currently?
   d. How often do you consume bushmeat in a month?
   e. What species do you consume?
2. Who else consumes bushmeat killed by ammunition in your family?
   a. (Do children also eat it?)
3. Can you tell me how you source/buy the meat?
4. Can you tell me how you prepare the meat?
   a. (Do you cook it? Can you describe how you cook the bushmeat?)
   b. What do you do to the part of the meat wounded by the bullet?
   c. (Do you clean the meat wounded by the bullet? Or discard it? How? Why?)

E. Feedback

1. Are there any other thing about these subjects that you would like to bring to our attention?
